# Supplementary material for: Reconciling Mining with the Conservation of Cave Biodiversity: A Quantitative Baseline to Help Establish Conservation Priorities
Source: PLoS One. 2016 Dec 20;11(12):e0168348. doi: 10.1371/journal.pone.0168348 (PMC5173368; doi:10.1371/journal.pone.0168348)
Supplement: S1 Dataset — (ZIP) [file pone.0168348.s002.zip › Taxa/Serra Norte/SN_2013/N4E/Lista N4E-41.pdf]

CAVIDADE N4E-0041

Classe	Ordem	Fam/Outros	Gên/Outros	Espécie	S-U	U-S	Zon.
Annelida	Oligochaeta			sp.		X	
Arachnida	Acari	Prostigmata		sp.		X	
Arachnida	Araneae	Araneidae		jovem	X	X	E
Arachnida	Araneae	Ctenidae		jovem		1	
Arachnida	Araneae	Ochyroceratidae	*Ochyrocera*	sp.		X	
Arachnida	Araneae	Ochyroceratidae	*Speocera*	sp.		X	
Arachnida	Araneae	Pholcidae	*Mesabolivar*	sp.		X	
Arachnida	Araneae	Theridiosomatidae	*Plato*	sp.1	X	X	P
Arachnida	Opiliones	Escadabiidae		sp.2		X	
Arachnida	Pseudoscorpiones	Chernetidae		sp.		X	
Chilopoda	Scolopendromorpha	Cryptopidae	*Cryptops*	sp.		X	
Entognatha	Collembola			sp.		X	
Insecta	Blattodea			jovem	1	X	E
Insecta	Coleoptera	Tenebrionidae	Alleculinae	sp.		X	
Insecta	Diptera	Cecidomyiidae		sp.		X	
Insecta	Diptera	Psychodidae	Psychodinae	sp.		X	
Insecta	Diptera	Psychodidae	*Psychodopygus*	*wellcomei*		X	
Insecta	Diptera	Psychodidae	*Sciopemyia*	*sordellii*		X	
Insecta	Diptera	Sciaridae		sp.	X		E
Insecta	Diptera	Tipulidae		sp.		X	
Insecta	Heteroptera	Reduviidae	*Ploiaria*	sp.		X	
Insecta	Heteroptera	Reduviidae	*Zelurus*	*festivus*		X	
Insecta	Homoptera	Cixiidae		sp.	X	X	E
Insecta	Hymenoptera	Formicidae	*Camponotus*	*atriceps*		X	
Insecta	Hymenoptera	Formicidae	*Pachycondyla*	*constricta*		X	
Insecta	Hymenoptera	Formicidae	*Pachycondyla*	sp.1	X		E
Insecta	Hymenoptera	Formicidae	*Pachycondyla*	sp.2	X		E
Insecta	Lepidoptera	Noctuoidea		sp.		X	
Insecta	Lepidoptera	Tineoidea		jovem		X	
Insecta	Orthoptera	Phalangopsidae	*Phalangopsis*	sp.	11		P
Insecta	Psocoptera	Epipsocidae	*Epipsocus*	sp.2	X		E/P
Insecta	Psocoptera	Liposcelididae	*Liposcelis*	sp.1	X	X	E
Insecta	Thysanura	Nicoletiidae	Nicoletiinae	sp.1		X	
Malacostraca	Isopoda	Armadillidae		sp.1		X	
Amphibia	Anura			sp.	1	7	E/P
Mammalia	Chiroptera	Emballonuridae		sp.	1		
